# Implementation of the DSM-5 and ICD-11 Dimensional Models of Maladaptive Personality Traits Into Pre-bariatric Assessment

**DOI:** 10.3389/fpsyg.2021.814421

**Published:** 2022-01-05

**Authors:** Karel D. Riegel, Judita Konecna, Martin Matoulek, Livia Rosova

**Affiliations:** ^1^Department of Addictology, 1^st^ Faculty of Medicine, Charles University and General University Hospital, Prague, Czechia; ^2^3^rd^ Department of Medicine—Department of Endocrinology and Metabolism, 1^st^ Faculty of Medicine, Charles University and General University Hospital, Prague, Czechia; ^3^Department of Psychiatry, 1^st^ Faculty of Medicine, Charles University and General University Hospital, Prague, Czechia; ^4^Department of Applied Mathematics and Statistics, Faculty of Mathematics, Physics and Informatics, Comenius University, Bratislava, Slovakia

**Keywords:** bariatric surgery, obesity, PID-5, AMPD, cluster analysis, personality trait, ICD-11

## Abstract

**Background:** Personality pathology does not have to be a contraindication to a bariatric surgery if a proper pre-surgical assessment is done. Indicating subgroups of patients with their specific needs could help tailor interventions and improve surgical treatment outcomes.

**Objectives:** Using the Alternative DSM-5 model for personality disorders (AMPD) and the ICD-11 model for PDs to detect subgroups of patients with obesity based on a specific constellation of maladaptive personality traits and the level of overall personality impairment.

**Methods:** 272 consecutively consented patients who underwent a standard pre-surgical psychological assessment. The majority were women (58.0%), age range was 22–79 years (*M* = 48.06, *SD* = 10.70). Patients’ average body mass index (BMI) was 43.95 kg/m^2^. All participants were administered the Personality Inventory for DSM-5 (PID-5) from which Level of Personality Functioning Scale-Self Report (LPFS-SR) and Standardized Assessment of Severity of Personality Disorder (SASPD) scores were gained using the “crosswalk” for common metric for self-reported severity of personality disorder. The k-means clustering method was used to define specific subgroups of patients with obesity and replicated for equality testing to the samples of non-clinical respondents and psychiatric patients.

**Results:** The cluster analysis detected specific groups in the sample of patients with obesity, which differed quantitatively from the samples of non-clinical respondents and psychiatric patients. A vast majority of patients with obesity showed above-average values in most of the PID-5 facets compared to the United States representative general community sample. In two out of the three clusters defined, patients demonstrated moderate (> *M* + 1.5 × *SD*) to severe (> *M* + 2.0 × *SD*) personality psychopathology within the Detachment and Negative Affectivity domains according to PID-5, which in one of the clusters corresponded to the mild overall impairment in both, LPFS-SR (*M* = 2.18, *SD* = 0.27) and SASPD (*M* = 8.44, *SD* = 2.38). Moreover, higher levels of psychopathology prove to be associated with higher age and use of psychiatric medication.

**Conclusions:** The dimensional DSM-5 and ICD-11 trait models are suitable procedures for defining specific “characters” of patients in a pre-bariatric setting. As such, they help to identify subgroups of patients with obesity who are different from general population and psychiatric patients. Implications for clinical practice and further research are discussed.

## Introduction

The level of personality pathology and mental disorders including personality disorders (PDs) is discussed in regard to obesity treatment, especially when the risks and profits of bariatric surgery are in question (Walfish et al., 2007; Chalopin et al., 2020). Some findings indicate a less successful outcome for obesity patients suffering from adjustment disorders, depression and/or PDs, compared to patients who are not mentally ill (Kinzl et al., 2006). Although the PD diagnosis is one of the three most frequently cited factors that negatively affect weight reduction after bariatric surgery (Livhits et al., 2012), the way personality pathology is reported prevents a systematic comparison of results. First, studies address personality variables by various tools, which may cause inconsistencies in the presented findings (Rowe et al., 2000; Guisado et al., 2002; Tsushima et al., 2004; De Panfilis et al., 2006; Leombruni et al., 2007). Another problem is that there is no clear consensus among psychologists as to whether (Larsen et al., 2004) and how to assess the overall personality psychopathology in pre-bariatric evaluations (Bauchowitz et al., 2005; Fabricatore et al., 2006).

There is already some evidence that the reliance on the categorical approach to the classification of psychological variables, in addition to the existence of methodological differences between studies, is likely to have contributed to the reports of inconsistent findings regarding the role of psychopathology on the bariatric treatment outcomes (Oltmanns et al., 2020). Pre-surgical use of dimensional assessment of the hierarchical model of psychopathology is important in predicting post-surgical outcomes, because it provides more statistical power to detect change through enhancing measurement reliability (Marek et al., 2016). The issue of reliability can be considered as one of the main reasons why the dimensional classification of personality and psychopathology is increasingly promoted (Kotov et al., 2017). To date, several studies have been published using the Minnesota Multiphasic Personality Inventory-2 (MMPI-2) and its restructured form (MMPI-2-RF) in bariatric settings (e.g., Tsushima et al., 2004; Marek et al., 2015a,b, 2017). The last of the cited studies provided strong supporting evidence that disinhibited and depressed personality along with the presence of borderline, antisocial, and narcissistic personality traits predicts less longitudinal body mass index (BMI) reduction 5 years after bariatric surgery (Marek et al., 2017). Similarly, borderline personality problems along with anxiety-related disorders and higher proneness to stress have been associated with less optimal BMI and weight outcomes 5 years after bariatric surgery in a recent study using the Personality Assessment Inventory (PAI) in a representative sample of bariatric patients (Oltmanns et al., 2020). Put together, using the dimensional assessment tools such as MMPI-2-RF and PAI supports the clinical utility of dimensional systems for pre-surgical personality assessment of bariatric patients. The Alternative DSM-5 model for personality disorders (AMPD) might be one of such systems.

The AMPD has been introduced in the 5th edition of the Diagnostic and Statistical Manual of Mental Disorders (DSM-5) section III as a dimensional alternative for measuring personality psychopathology to the current categorical classification maintained in DSM-5 section II [American Psychiatric Association [APA], 2013]. The key innovation of the AMPD is defining PDs on the basis of impairments in personality functioning (criterion A) and the presence of maladaptive personality traits (criterion B). This makes it possible to create a more plastic and vivid image of a personality than simply reaching a diagnostic threshold for the presence or absence of a PD diagnosis by meeting a limited number of criteria. Moreover, the AMPD approach to PDs is largely commensurate with the 11th edition of the International Classification of Diseases (ICD-11) [World Health Organization [WHO], 2019], which also adopts a dimensional approach to the classification of PDs that focuses on global level of severity and five trait qualifiers. In both classifications trait domains are used as specifiers that contribute to the individual expression of personality disturbance in addition to the overall classification of severity (e.g., mild, moderate, or severe) (Zimmermann et al., 2019). Both ICD-11 and AMPD describe trait domains of Negative Affectivity, Detachment, Antagonism/Dissociality, and Disinhibition. In addition, the AMPD also includes a separate domain of Psychoticism, whereas the ICD-11 includes a separate domain of Anankastia. Furthermore, the 25 trait facets in the AMPD model may provide more detailed description of the subtle nuances of the patient’s personality (Bach and First, 2018), which can be enriching also for the ICD-11’s five trait domains. The high complementarity of the two classifications makes it possible to use uniform instruments for this purpose, for example the Personality Inventory for DSM-5 (PID-5), a self-assessment tool designed to directly evaluate the proposed system of personality traits in the AMPD.

Since its release in 2013, the AMPD has stimulated extensive research with promising findings (Zimmermann et al., 2019), which go well beyond the scope of this article. However, with regard to some broadband tools for assessing personality psychopathology in bariatric settings, we consider it important to mention some conclusions regarding the convergent validity of the AMPD. Previous research has shown significant convergence between the Personality Psychopathology-Five (PSY-5) domains of MMPI-2-RF and the domains and facets of the PID-5 (Anderson et al., 2013). Moreover, there is also empirical evidence about the substantial convergence between the DSM-5 pathological traits and a range of clinical issues as instantiated in the PAI (Hopwood et al., 2013). The broad convergence between personality and psychopathology constructs is consistent with the hypothesis that the higher-order domains that describe covariation in normal personality, personality disorder, and clinical constructs more generally, might be thought of as psychological systems, as might be seen e.g., in the Research Domain Criteria (RDoC) (Insel et al., 2010) or the Hierarchical Taxonomy of Psychopathology (HiTOP) (Kotov et al., 2017) framework.

To date, some studies (e.g., Marek et al., 2015b,^2016^) have explained the pre-surgical psychological risk factors for poor bariatric surgery outcomes associated with PSY-5 on basis of the RDoC, but to the best of our knowledge, there is only one study using tools designed for the AMPD in patients with obesity. Its authors demonstrated a specific constellation of maladaptive personality traits of emotional lability, anhedonia, impulsivity and depressivity in a relatively small sample (*n* = 55) of obese female patients with a binge eating disorder (BED) using a forward stepwise linear regression analysis (Aloi et al., 2020). The authors conclude that these preliminary findings could be beneficial in clinical practice where specialists should evaluate the presence of specific personological traits to develop specific therapeutic approaches to offer a tailor-made treatment for patients with comorbid obesity and BED.

It has been pointed out (Gerlach et al., 2015), that the best way how to treat obesity is to make the treatment as individualized as possible, which seems particularly meaningful for patients with a more severe personality psychopathology (Kinzl et al., 2006). Following the previous research focused on the dimensional evaluation of psychopathology in patients with obesity (Aloi et al., 2020) and its importance for the prediction of bariatric outcomes (Marek et al., 2015b; Oltmanns et al., 2020), the first goal of this study is to use PID-5 to detect subgroups of patients based on a specific constellation of maladaptive personality traits. As the *a priori* internal structure of the PID-5 is not taken into account, methods such as confirmatory factor analysis are not applicable. Also, feature extraction methods are most commonly used to a different purpose, i.e., simplification of the set of variables. For these reasons we found cluster analysis to be the most suitable approach. We assume that, based on a cluster analysis, we will be able to detect several clusters in a sample of patients with obesity which will be quantitatively different from the general population sample and the sample of psychiatric patients. Given the fact that the ICD-11 is the only authoritative nomenclature for all WHO countries, while the AMPD remains an “alternative” to the existing categorical model retained in DSM-5 Section II, we applied cluster analysis not only to the AMPD, but also the ICD-11 trait model for PDs. Although the specific constellation of personality traits may not in itself indicate the overall degree of personality impairment, previous findings have shown that the PID-5 score is consistent with various measures of personality functioning (Zimmermann et al., 2019). Therefore, as the second goal of the study, we use a crosswalk between PID-5 and the Level of Personality Functioning Scale-Self Report (LPFS-SR) and the Standardized Assessment of Severity of Personality Disorder (SASPD) scores (Zimmermann et al., 2020) to verify differences in the severity of personality psychopathology between individual clusters. Consequently, we hypothesize that based on the AMPD criteria, we will be able to detect groups of patients with obesity with different care needs, which may have a significant prospective impact on the effectiveness of obesity treatment.

## Materials and Methods

### Participants

Three samples were used in the study. Sample 1 was composed of patients with obesity, Sample 2 contained university students from various fields of study, working volunteers and pensioners, and Sample 3 was composed of psychiatric patients. Samples 2 and 3 were used to compare the equality of the cluster analysis with Sample 1. To be included in a sample, all participants had to be over 18 years of age. Participation in the study was voluntary and anonymous for all respondents. Participants were not rewarded for their participation in the study. As seen in the Plan of analysis section, we used the PID-5 Response Inconsistency Scale (PID-5-RIS) for removing participants with invalid data. The number of removed participants within the samples was as follows: Sample 1 *n* = 80; Sample 2 *n* = 37; Sample 3 *n* = 37.

Sample 1 consisted of patients with severe obesity (*n* = 272; average BMI was 43.95 kg/m^2^). Patients were consecutively recruited from both the outpatient and inpatient units of the Department of Endocrinology and Metabolism of the General University Hospital in Prague. These individuals were considered as potential candidates for a bariatric surgery between 1/2017 and 3/2020. The exclusion criteria were: age < 18 years, BMI < 30.10 kg/m^2^, a neurological condition or acute psychotic illness that could affect cognitive functioning, presence of type 1 diabetes mellitus. Gender representation was almost balanced: women slightly prevailed over men (*n* = 159, 58.0%). The age range was 22–79 years (*M* = 48.06, *SD* = 10.70). Distribution according to the highest attained level of education was as follows: primary education 4.78%, secondary education 57.35%, some college 4.04%, university degree 25.74%, not specified 8.09%.

Sample 2 consisted of respondents from the general population included in the international study by Bach et al. (2020) (*n* = 335). Gender representation was not balanced: women prevailed over men (*n* = 217, 64.8%). The age range was 18–84 years (*M* = 32.07, *SD* = 12.81). Distribution according to the highest attained level of education in this group was as follows: primary education 1.2%, secondary education 53.4%, some college 6.3%, undergraduate degree 15.8%, graduate degree 23.3%.

Sample 3 consisted of psychiatric patients included in the study by Riegel et al. (2018) (*n* = 106). Gender representation was not balanced: again, women prevailed over men (*n* = 70, 66.0%). The age range was 18–65 years (*M* = 36.58, *SD* = 11.53). Distribution according to the highest attained level of education in this group was as follows: primary education 11.3%, secondary education 65.1%, some college 6.6%, undergraduate degree 0.9%, graduate degree 16.0%.

### Procedures

Each patient from Sample 1 was examined by a multidisciplinary team consisting of a surgeon, gastroenterologist, dietician, and clinical psychologist. As part of a standard psychological examination, all subjects enrolled in this study were asked to fill the PID-5 and sociodemographic questionnaires either online after the assignment *via* a unique identification code, or by the paper-and-pencil method. Potential differences between the types of administration were assessed by comparing all of the mean scores. As no statistically significant differences were found, the subgroups were merged. Only complete and valid PID-5 protocols were included in the analysis (see Plan of analysis). Prior to the evaluation, patients were informed about the objective of the study and asked to consent to having their medical and psychological data used for research purposes. The study protocol and the informed consent form were approved by the ethics committee of the General University Hospital in Prague.

### Measures

#### Sociodemographic Questionnaire

All respondents were asked to answer questions regarding their age, weight, height, gender, level of education, presence of type 2 diabetes mellitus, history of bariatric surgery, and use of psychiatric medication.

#### Personality Inventory for DSM-5

The PID-5 (Krueger et al., 2012) is a self-administered of 220 items that measures 25 personality trait facets according to the criterion B of the AMPD. The facets are grouped into five broad domains: Negative Affectivity, Detachment, Antagonism, Disinhibition, and Psychoticism. Participants are asked to evaluate each item on a Likert scale ranging from 0 (“very untrue or often untrue”) to 3 (“very true or often true”). Higher average scores indicate greater dysfunction in a specific facet or domain. In the present study, in Sample 1, the Cronbach’s alphas for all 25 facets ranged from 0.56 (suspiciousness) to 0.94 (eccentricity) indicating good internal consistency. For the purpose of comparing the PID-5 scores with LPFS-SR and SASPD we used an algorithm which has been recently developed to evaluate the combined AMPD and the ICD-11 personality traits model based on six higher-order domains (i.e., Negative affectivity, Detachment, Antagonism, Disinhibition, Anankastia and Psychoticism), covering 17 of the lower-order facets and featuring a total number of 34 items. This algorithm is captured by the Personality Inventory for DSM-5-Brief Form Plus (PID-5BF+) (Kerber et al., 2020).

#### Level of Personality Functioning Scale-Self Report

The LPFS-SR (Morey, 2017) is a comprehensive self-report measure for assessing criterion A of the AMPD. It features descriptions of five different levels of impairment in the domains of identity, self-direction, empathy, and intimacy. It includes 80 items that are rated on four-point Likert scales ranging from 1 (“totally false, not at all true”) to 4 (“very true”). Since the LPFS-SR was not administered to patients, as the evaluation of the general personality impairment was not an inherent part of pre-bariatric assessment, we have worked—for the purposes of this study—exclusively with the total (average) scores based on the simplified scoring scheme proposed by Zimmermann et al. (2020).

#### Standardized Assessment of Severity of Personality Disorder

The SASPD (Olajide et al., 2018) is a self-report measure that provides an index of PD severity. It includes 9 items that are rated using 0–3 response options with unique descriptions and captures 9 distinct PD features, which are separately rated in terms of severity. As in the case of LPFS-SR, we’ve also worked for SASPD exclusively with the total (sum) scores based on the scoring scheme proposed by Zimmermann et al. (2020).

### Plan of Analysis

To ensure the validity of data in all three samples, we used the PID-5-RIS developed by Keeley et al. (2016), which has proven successful in detecting random responses in the original version of PID-5 and has been verified by a number of recent studies (Bagby and Sellbom, 2018; Somma et al., 2018; Lowmaster et al., 2020). In line with these studies, we excluded respondents with a PID-5-RIS score ≥ 17. Subsequently, the internal consistency of each trait was examined by calculating Cronbach’s alpha.

Afterward, based on the measured patient scores, subgroups were formed which associated patients with similar scores. The division was performed using the well-known k-means clustering method, in which *n* observations are divided into *k* clusters, where each observation belongs to the cluster with the closest mean (cluster center). The number of clusters was chosen using the silhouette method (based on how much a point is similar to its own cluster compared to other clusters) supported by the elbow method (based on the average distance of each point in a cluster to its cluster’s center). One-way ANOVA was used to analyze whether the scores of the three clusters differed significantly.

In the next step the algorithm developed by Bach et al. (2017) was used to derive ICD-11 trait domains using PID-5 trait facet scores. Finally, relationships between the established clusters and the sociodemographic data were examined through the cross-tabulation analysis.

To obtain information about the potential results of the LPFS-SR and SASPD questionnaires, we used a crosswalk table linking total scores of different measures (Zimmermann et al., 2020). Because this connection is made only through PID-5BF+ we first needed to abbreviate the full 220-item PID-5 following the algorithm proposed by Kerber et al. (2020).

The statistical program R 4.1.0 (R Core Team, 2021) was used in the analysis.

## Results

### Defining Trait-Based Personality Clusters

**FIGURE 1:**
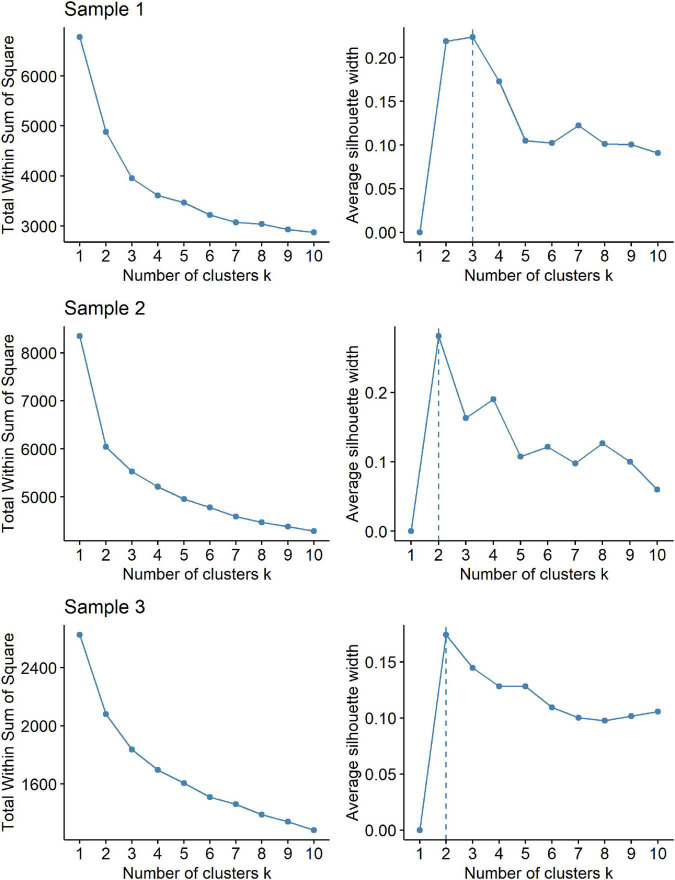
Elbow diagram (left) and silhouette diagram (right) for all three datasets—patients with obesity (Sample 1), respondents from the general population (Sample 2) and psychiatric patients (Sample 3).

Figure 1 shows the elbow diagram and the silhouette diagram for all three datasets. For Samples 2 and 3 a division into two clusters is proposed, while three clusters are recommended as the optimal solution for the examined Sample 1. In the following paragraphs, we refer to the results relating exclusively to Sample 1.

**FIGURE 2:**
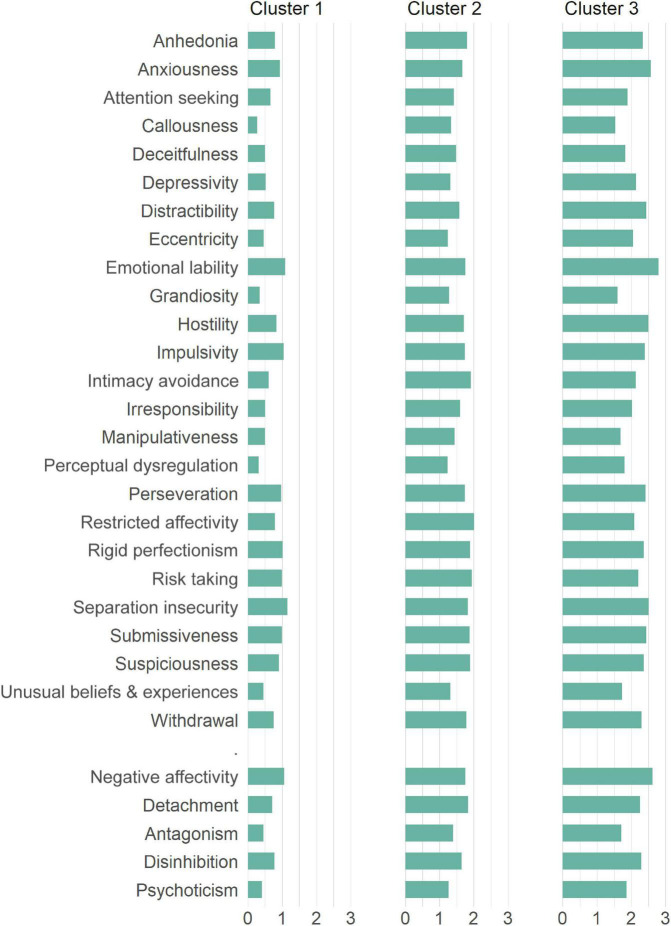
The average PID-5 facet and domain scores within clusters for patients with obesity (Sample 1).

Table 1 and Figure 2 provide more detailed information about the average PID-5 facet scores and the corresponding standard deviations across all clusters. Clusters differed by the number of participants. The lowest number of respondents after the division into three clusters was in Cluster 1 (*n* = 20, 7.4%). In this cluster, the lowest average values of the PID-5 facet scores were achieved in the range from *M* = 0.27 (*SD* = 0.23) for callousness to *M* = 1.15 (*SD* = 0.70) for separation insecurity. Cluster 3 (*n* = 107, 39.3%) was the cluster with the highest values of the average score, which ranged from *M* = 1.54 (*SD* = 0.38) for callousness to *M* = 2.79 (*SD* = 0.63) for emotional lability. Cluster 2 comprised the highest number of respondents (*n* = 145, 53.3%) and its average score values ranged from *M* = 1.23 (*SD* = 0.21) for perceptual dysregulation to *M* = 2.01 (*SD* = 0.48) for restricted affectivity. In all cases, the hypothesis of equality of the scores of the three clusters was tested and in all cases it was rejected (*p* < 0.05).

**TABLE 1:** Cronbach’s alphas, average scores and standard deviations of PID-5 facets and domains within clusters.

Facet/domain			Clusters					
			1 (*n* = 20)		2 (*n* = 145)		3 (*n* = 107)	
	No. items	Cr. alpha	*M*	*SD*	*M*	*SD*	*M*	*SD*
Anhedonia	8	0.82	0.79	0.51	*1.81*	0.35	***2.33***	0.43
Anxiousness	9	0.89	0.94	0.55	1.67	0.44	***2.57***	0.64
Attention seeking	8	0.88	0.66	0.81	1.41	0.49	***1.89***	0.75
Callousness	14	0.75	0.27	0.23	***1.34***	0.20	***1.54***	0.38
Deceitfulness	10	0.81	0.50	0.56	***1.47***	0.33	***1.83***	0.50
Depressivity	14	0.92	0.52	0.51	*1.32*	0.28	***2.14***	0.67
Distractibility	9	0.88	0.77	0.71	*1.57*	0.46	***2.44***	0.65
Eccentricity	13	0.94	0.46	0.53	1.25	0.35	***2.05***	0.68
Emotional lability	7	0.86	1.09	0.67	*1.74*	0.50	***2.79***	0.63
Grandiosity	6	0.77	0.35	0.46	1.28	0.37	*1.60*	0.59
Hostility	10	0.82	0.84	0.53	*1.71*	0.40	***2.50***	0.47
Impulsivity	6	0.76	1.04	0.61	***1.74***	0.53	***2.40***	0.60
Intimacy avoidance	6	0.70	0.61	0.64	***1.91***	0.51	***2.13***	0.52
Irresponsibility	7	0.72	0.51	0.40	***1.60***	0.27	***2.02***	0.47
Manipulativeness	5	0.65	0.50	0.52	1.44	0.44	*1.69*	0.56
Perceptual dysregulation	12	0.81	0.32	0.25	***1.23***	0.21	***1.81***	0.49
Perseveration	9	0.77	0.97	0.56	*1.74*	0.39	***2.42***	0.46
Restricted affectivity	7	0.58	0.79	0.44	***2.01***	0.48	***2.08***	0.54
Rigid perfectionism	10	0.86	1.02	0.66	*1.89*	0.52	***2.36***	0.63
Risk taking	14	0.82	0.99	0.40	***1.94***	0.29	***2.21***	0.27
Separation insecurity	7	0.81	1.15	0.70	*1.82*	0.56	***2.51***	0.68
Submissiveness	4	0.70	0.99	0.68	*1.87*	0.57	***2.44***	0.66
Suspiciousness	7	0.56	0.91	0.36	***1.89***	0.40	***2.37***	0.42
Unusual beliefs and experiences	8	0.81	0.46	0.50	*1.32*	0.38	***1.73***	0.63
Withdrawal	10	0.84	0.76	0.37	*1.78*	0.52	***2.30***	0.60
Negative Affectivity			1.06	0.54	***1.74***	0.37	***2.62***	0.47
Detachment			0.72	0.40	***1.83***	0.34	***2.25***	0.38
Antagonism			0.45	0.44	***1.40***	0.31	***1.71***	0.46
Disinhibition			0.77	0.49	***1.64***	0.31	***2.29***	0.39
Psychoticism			0.41	0.33	*1.27*	0.24	***1.86***	0.50
NEGATIVE AFFECTIVITY			0.85	0.49	1.58	0.31	2.50	0.49
DETACHMENT			0.72	0.39	1.90	0.38	2.17	0.41
DISSOCIALITY			0.49	0.36	1.44	0.24	1.83	0.36
DISINHIBITION			0.83	0.39	1.71	0.26	2.27	0.31
ANANKASTIA			0.99	0.51	1.81	0.36	2.39	0.42

*Scores above M + 1.0 × SD (normative United States values) are italicized; scores above M + 1.5 × SD are italicized and bolded; scores above M + 2.0 × SD are italicized, bolded and underlined. ICD-11 trait domains are capitalized.*

As can be seen in the PID-5 domain values in Figure 2 and Table 1, the lowest value in Cluster 1 was acquired by the Psychoticism domain (*M* = 0.41, *SD* = 0.33) while the Negative Affectivity domain had the highest value (*M* = 1.06, *SD* = 0.54). This is similar to Cluster 3, where the highest value was also Negative Affectivity (*M* = 2.62, *SD* = 0.47), however, the lowest was Antagonism (*M* = 1.71, *SD* = 0.46). Cluster 2 differed in the highest value achieved by Detachment (*M* = 1.83, *SD* = 0.34), with the lowest value being analogous to Cluster 1 with Psychoticism (*M* = 1.27, *SD* = 0.24). As with the individual facet scores testing, equality between cluster scores was not confirmed for domains.

**FIGURE 3:**
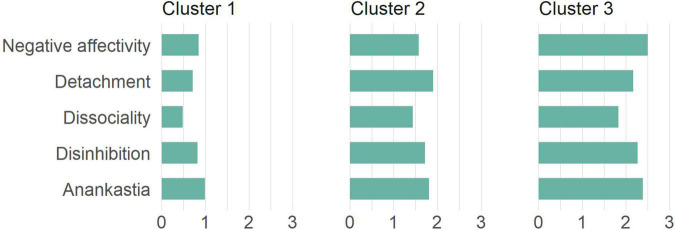
The average ICD-11 trait domain scores within clusters for patients with obesity (Sample 1).

From the perspective of the ICD-11 domain scores, the lowest value in Cluster 1 was obtained by the Dissociality domain (*M* = 0.49, *SD* = 0.36) and the highest by Anankastia (*M* = 0.99, *SD* = 0.51). In Cluster 2 was also the lowest value Dissociality (*M* = 1.44, *SD* = 0.24), however, the highest value was acquired by the Detachment domain (*M* = 1.90, *SD* = 0.38). In line with both previous clusters, Dissociality was the lowest domain also in Cluster 3 (*M* = 1.83, *SD* = 0.36) while the Negative Affectivity domain had the highest value (*M* = 2.50, *SD* = 0.49). For more details we refer to Table 1 and Figure 3.

Due to the absence of representative Czechia normative values of the PID-5, we compared the average scores across the clusters with the normative data from the United States representative sample (Krueger et al., 2012). This comparison helped us in defining the most clinically significant features within the clusters based on the height of the standard deviations for each individual facet and domain. In Cluster 1, none of the facets or domains exceeded the value of the normative score mean plus one standard deviation (*M* + 1.0 × *SD*). In Cluster 2, 20 out of 25 PID-5 facets and all five domains scored > *M* + 1.0 × *SD*, of which eight facets and four domains scored > *M* + 1.5 × *SD* and 1 facet scored > *M* + 2.0 × *SD*. In Cluster 3, all 25 facets and all five domains exceeded *M* + 1.0 × *SD*, of which seven facets scored > *M* + 1.5 × *SD* and 16 facets and all five domains scored > *M* + 2.0 × *SD*. Detailed information about the distribution of the facet and domain scores are provided in Table 1.

### Associations Between Clusters and Sociodemographic Data

From the perspective of age, the lowest average age (*M* = 41.22, *SD* = 12.45) was observed in Cluster 1, followed by Cluster 3 (*M* = 47.66, *SD* = 9.50) and Cluster 2 (*M* = 49.23, *SD* = 11.03). The male-female ratio was almost balanced in Cluster 1 (47.4% vs. 52.6%) and Cluster 2 (44.8% vs. 55.2%), whereas in Cluster 3 women predominated over men (35.5% vs. 64.5%). From the perspective of psychiatric medication use, the proportions of patients between clusters were unbalanced: Cluster 1 (10.0%); Cluster 2 (28.3%); and Cluster 3 (47.7%). The average BMI was almost balanced between the clusters: Cluster 1 (*M* = 45.46, *SD* = 12.24); Cluster 2 (*M* = 44.49, *SD* = 8.60); and Cluster 3 (*M* = 43.72, *SD* = 7.89). The differences between clusters and sociodemographic data were not statistically significant, except for age (*F* = 4.72, *df* = 2, *p* = 0.0097) and the use of psychiatric medication (χ^2^ = 15.99, *df* = 2, *p* < 0.05).

### Matching the Clusters With the Level of Overall Psychopathology

After the transformation of PID-5 to PID-5BF+, the cluster with the lowest acquired values of average facet scores became Cluster 2 (except for intimacy avoidance and withdrawal, both contained the lowest facet values). Subsequently, this was reflected in the average value of LPFS-SR, which was *M* = 1.79 (*SD* = 0.37) for Cluster 1, *M* = 1.63 (*SD* = 0.22) for Cluster 2, and *M* = 2.18 (*SD* = 0.27) for Cluster 3 and SASPD, which was *M* = 4.75 (*SD* = 3.45) for Cluster 1, *M* = 3.48 (*SD* = 2.17) for Cluster 2, and *M* = 8.44 (*SD* = 2.38) for Cluster 3. Detailed PID-5BF+ facet scores for each cluster are provided in Table 2.

**TABLE 2:** Average scores and standard deviations of PID-5BF+ facets within clusters.

Facet	Clusters					
	1 (*n* = 20)		2 (*n* = 145)		3 (*n* = 107)	
	*M*	*SD*	*M*	*SD*	*M*	*SD*
Anhedonia	0.68	0.63	0.61	0.69	1.31	0.87
Anxiety	0.52	0.60	0.48	0.68	1.48	1.01
Deceitfulness	0.82	0.89	0.57	0.65	1.11	0.84
Distractibility	0.68	0.67	0.57	0.68	1.47	0.82
Eccentricity	0.58	0.75	0.24	0.43	0.93	0.82
Emotional lability	1.32	0.83	0.67	0.67	1.85	0.79
Grandiosity	0.35	0.56	0.23	0.45	0.55	0.69
Impulsivity	0.95	0.84	0.80	0.73	1.60	0.81
Intimacy avoidance	0.45	0.67	0.53	0.69	0.76	0.78
Irresponsibility	0.55	0.63	0.27	0.44	0.81	0.76
Manipulativeness	0.28	0.47	0.18	0.34	0.37	0.56
Perceptual dysregulation	0.15	0.40	0.15	0.36	0.52	0.69
Perseveration	0.68	0.67	0.50	0.57	1.16	0.69
Rigid perfectionism	0.95	0.78	0.77	0.67	1.25	0.83
Separation insecurity	0.85	0.75	0.59	0.64	1.35	0.78
Unusual beliefs and experiences	0.75	0.90	0.35	0.57	0.96	0.85
Withdrawal	0.62	0.58	0.65	0.71	1.29	0.75

## Discussion

In line with the first goal of the current investigation based on the k-means clustering method, we defined three clusters with a specific distribution of the AMPD maladaptive personality traits and varying degrees of psychopathology within Sample 1. As the three-cluster solution did not prove to be optimal in the remaining Samples 2 and 3, we can assume that the more heterogeneous sample of patients with obesity contains certain specifics that allow for more precise definition of personality indicators than the mere distinction between greater and lesser severity, which proved typical for more homogeneous samples of non-clinical respondents and psychiatric patients. On the one hand, the difference between Clusters 1 and 2 in Sample 1 underlines to some extent the fact that obesity is a multifactorial physical health problem, which is primarily a consequence of a sustained positive energy balance (Gerlach et al., 2015) to which psychological factors (such as personality variables) of an individual may or may not contribute. On the other hand, the difference between Clusters 2 and 3 suggests that another specific group of individuals can be detected among obese patients with higher psychopathology, whose personality variables significantly interfere with adaptive psychological functioning. This is confirmed not only by the prescription of psychiatric medication in almost 50% of respondents from Cluster 3, but also by the degree of overall personality impairment according to the SASPD in this cluster. Considering that the relationship between the degree of personality impairment, the use of psychiatric medication and obesity has been already pointed out (Frankenburg and Zanarini, 2006), the distinction of Sample 1 into three specific clusters has significant clinical justification, especially in relation to more serious personality problems such as borderline psychopathology.

Although the clusters within the Sample 1 proved to be mutually unequal from the quantitative point of view, we are aware that from the perspective of clinical practice, qualitative differences in facet/domain distribution between the clusters have informative value only when tested for their clinical significance. Interestingly, 93% of respondents in Sample 1 were included in clusters with above-average mean values of the majority of PID-5 facets and domains in comparison to the values of a representative United States sample. This result is in line with some previous studies which have shown that the incidence of psychiatric disorders, including personality disorders, increases in patients with obesity (Baumeister and Härter, 2007; Petry et al., 2008). The only exception were the respondents included in Cluster 1, who did not show above-average values in any of the PID-5 facets and domains. Reflecting that it was a cluster with a distinctly lower number of patients who were in addition significantly younger compared to the other two clusters, the lower proportion of maladaptive personality traits observed in this cluster can be seen as an exception rather than a rule, considering our Sample 1 as a whole, in which higher age seems to be connected with higher psychopathology. Although it can be expected that patients belonging to this cluster may cooperate well in both the pre-surgical and post-surgical phase of bariatric treatment without the need to undergo any additional psychological intervention, a somewhat higher proneness to the Negative Affectivity traits of separation insecurity and emotional lability should always be investigated in the course of a pre-bariatric psychological evaluation. Interestingly, when the algorithm for deriving ICD-11 domains was applied, the Anankastia domain became the most prominent trait domain in this cluster. We assume, that was probably due to the omission of the separation insecurity facet, which is not included in a triplet of PID-5 facets primarily defining Negative Affectivity domain according to this algorithm (Bach et al., 2017). In this case, it seems desirable to confirm the role of the Anankastia domain in Cluster 1 *via* more specific tools for the ICD-11 trait model, such as Personality Inventory for ICD-11 (PiCD) (Oltmanns and Widiger, 2018).

In the case of Cluster 2, the restricted affectivity, intimacy avoidance, risk taking, suspiciousness, rigid perfectionism, and irresponsibility facets deserve special attention. In regard to specific PDs according to the AMPD (American Psychiatric Association [APA], 2013), this constellation of personality traits most corresponds to the criteria of an obsessive-compulsive personality disorder, in which compulsivity for repetitive behavior despite its negative consequences is a central feature. Behavioral patterns of compulsive eating are common across several eating-related conditions (Moore et al., 2017). Kakoschke et al. (2019) indicated some evidence of deficits across the compulsivity-related cognitive processes among individuals with excessive eating-related problems. According to their findings, there were differences in terms of the valence of impaired reversal learning in patients with obesity and those with comorbid obesity and BED. While obese individuals without BED may be more likely to avoid responding based on previously punished behaviors, an increased sensitivity to rewards, and enhanced risk taking in relation to reward expectation might be common features in obese individuals with BED. Those patients’ risk-taking behavior in the reward domain shows similarities to substance use disorders (Voon et al., 2015). This distinction may lead to a consideration of the presence of two subtypes of patients with a predominance of obsessive-compulsive traits within Cluster 2, which is to some extent supported by the distribution of domains according to ICD-11. While the first subtype would be represented by patients with a predominance of Detachment and Anankastia domains, the second subtype would be represented by patients with a predominance of Detachment and Disinhibition domains. In terms of clinical implications, different pre-surgical interventions should be considered for both the subtypes of patients. While obese patients with concomitantly increased Anankastia might benefit from dieticians’ educational programs, in some cases supplemented by specific psychological interventions decreasing their anxiety about the surgery, in case of patients with increased Disinhibition, treatment approaches should seek to use explicit knowledge of the contingencies between actions and outcomes to update the maladaptive eating behavior, especially when comorbid eating disorder such BED is in question (Heriseanu et al., 2020). These interventions may be reminiscent of those used in the treatment of substance use disorders and should precede bariatric considerations.

Finally, in Cluster 3, clinically significant scores of *M* + 2 × *SD* in the emotional lability, anxiousness, separation insecurity, depressivity, impulsivity, risk taking and hostility facets—i.e., all of the PID-5 facets defining borderline personality disorder (BPD) within the AMPD (American Psychiatric Association [APA], 2013)—support the previous findings about the prevalence of borderline personality symptomatology among gastric surgery patients (Sansone et al., 2008). In addition, the degree of psychopathology in this cluster appears to be related to the significantly higher proportion of patients taking psychiatric medication in comparison to the other two groups. More recently, borderline personality problems, namely problems with the self and identity, and rocky and unstable interpersonal relationships, have been associated with higher 5-year outcomes in both BMI and weight after a bariatric surgery (Oltmanns et al., 2020). These findings are in line with another predictive study published by Marek et al. (2017), in which borderline features connected with behavioral/externalizing dysfunction, such as disinhibition and aggression, were one of the predictors responsible for a higher BMI at the 5-year outcome after a bariatric surgery. In this regard, our results provide further evidence of the clinical utility of dimensional tools for assessing personality traits such as PAI, MMPI-2-RF, and now also PID-5, in detecting borderline psychopathology in obesity patients. Early detection of borderline features in bariatric candidates might be crucial for treatment planning. Although there is already some evidence that mental health treatment after bariatric surgery influences short-term outcomes (Shen et al., 2016), in case of patients with more severe mental health problems such as BPD, long-term success of therapy is likely to be impeded given their personality structure (Gerlach et al., 2015). Thus, in such cases, specific treatment options focused on strengthening self-control skills should be applied not only after, but also before the bariatric surgery.

As the severity of impairment in the areas of self and interpersonal functioning is considered to be a core of personality psychopathology in the AMPD (Pincus et al., 2020) as well as in the ICD-11 model for PDs [Morey, 2017; Bach and First, 2018; World Health Organization [WHO], 2019], we tried—in line with the second goal of our investigation—to examine the overall level of personality impairment across the defined clusters *via* LPFS-SR and SASPD based on the PID-5BF+ scores. Although the averaging of psychopathology in Sample 1 hypothetically corresponded to Kernberg’s model of personality psychopathology (Kernberg, 1984), since Cluster 1 resembled normal, Cluster 2 neurotic, and Cluster 3 a borderline personality organization, this assumption was partially confirmed by LPFS-SR and SASPD only for Cluster 3, in which the trait-based borderline psychopathology corresponded with a mild impairment in the personality functioning, which is considered as the minimal threshold for yielding a Personality Disorder diagnosis according the ICD-11 [Bach and First, 2018; World Health Organization [WHO], 2019]. It could be assumed that although PID-5BF+ is a good screening tool for distinguishing between mild and more severe psychopathology, a 34-item measure cannot provide the diagnostic precision and coverage of a 220-item measure, especially with respect to the facet traits that are assessed with only two items (Kerber et al., 2020).

Our findings need to be considered with respect to certain limitations that may inspire future research. First of all, it is necessary to take into account the subjectivity of the respondents’ statements as PID-5 is a tool based on self-assessment. Moreover, from the perspective of the AMPD and the ICD-11 model for PDs as a whole, we consider the way of assessing overall personality impairment on the basis of the crosswalk between PID-5 and LPFS-SR and SASPD as another significant limit of the presented study. Further research would benefit from the inclusion of clinician-guided structured interviews focused on the AMPD criterion A, such as the Structured Clinical Interview for the DSM-5 AMPD (Clarkin et al., 2020), or at least from the administration of the LPFS-SR as a stand-alone measure. With regard the ICD-11 trait model, employment of the instruments specifically developed for ICD-11 as PiCD could provide further verification of our findings based on the PID-5. Another limitation might be seen in using representative United States norms as thresholds of clinical significance. Although the previous research has shown significant differences between the average PID-5 scores of the Czechia and American populations (Riegel et al., 2017), it should be borne in mind that this data were not obtained from a representative Czechia sample and thus their use could significantly underestimate the severity of psychopathology. With regard to the proposed treatment implications, another limit of the study can be seen in the absence of specific tools for the diagnosis of BED and a confirmation of obsessive-compulsive and borderline psychopathology in our Sample 1. Future research in this regard could provide important information on whether our hypothesis of the two subtypes of patients in our largest Cluster 2 is justified. In addition, our analysis did not aim to find specific score patterns for bariatric patients, which also creates room for a future study focusing on a variable-centered approach. Finally, we consider it important to mention that with regard to the cross-sectional study design, our results have only a limited predictive value. In this respect, we consider the presented research to be exploratory. Nevertheless, our study can be deemed an important first step for future confirmatory studies on a longitudinal basis.

## Conclusion

Overall, in line with the previous studies (Marek et al., 2015b,^2017^; Oltmanns et al., 2020), the current investigation has provided yet more support for the utility of using empirically-grounded, dimensional psychological assessments in pre-surgical evaluation. In contrast to the study by Larsen et al. (2004) and in line with study by Gerlach et al. (2015), our findings suggest a personality assessment to be a valuable procedure for delineation of specific “characters” of the bariatric patients that can provide important clinical information for tailoring obesity treatment planning. PID-5 seems to be a reliable instrument for identifying different groups of patients with obesity, which are quantitatively different from general community individuals and psychiatric patients. Our results support the effectiveness of the dimensional AMPD and ICD-11 models of maladaptive personality traits in terms of the distinction between none, mild and more severe personality pathologies within the population of bariatric candidates, and as such provide further evidence about the clinical utility of the AMPD and ICD-11 model for PDs outside of a standard psychiatric setting.

## Data Availability Statement

The raw data supporting the conclusions of this article will be made available by the authors, without undue reservation.

## Ethics Statement

The studies involving human participants were reviewed and approved by Ethics committee of the General University Hospital in Prague. The patients/participants provided their written informed consent to participate in this study.

## Author Contributions

KDR drafted the manuscript and was responsible for the final version of the manuscript. JK and MM were responsible for the data collection. LR conducted all the data analyses. All authors have read and approved the manuscript.

## Conflict of Interest

The authors declare that the research was conducted in the absence of any commercial or financial relationships that could be construed as a potential conflict of interest.

## Publisher’s Note

All claims expressed in this article are solely those of the authors and do not necessarily represent those of their affiliated organizations, or those of the publisher, the editors and the reviewers. Any product that may be evaluated in this article, or claim that may be made by its manufacturer, is not guaranteed or endorsed by the publisher.

## References

[B1] Aloi M. Rania M. Caroleo M. Carbone E. A. Fazia G. Calabrò G. (2020). How are early maladaptive schemas and DSM-5 personality traits associated with the severity of binge eating? *J. Clin. Psychol*. 76 539–548. 10.1002/jclp.22900 31733127

[B2] American Psychiatric Association [APA] (2013). *Diagnostic and Statistical Manual of Mental Disorders*, 5th Edn. Arlington: American Psychiatric Association.

[B3] Anderson J. L. Sellbom M. Bagby R. M. Quilty L. C. Veltri C. O. Markon K. E. (2013). On the convergence between PSY-5 domains and PID-5 domains and facets: implications for assessment of DSM-5 personality traits. *Assessment* 20 286–294. 10.1177/1073191112471141 23297369

[B4] Bach B. First M. B. (2018). Application of the ICD-11 classification of personality disorders. *BMC Psychiatry* 18:351. 10.1186/s12888-018-1908-3 30373564 PMC6206910

[B5] Bach B. Kerber A. Aluja A. Bastiaens T. Keeley J. W. Claes L. (2020). International assessment of DSM-5 and ICD-11 personality disorder traits: toward a common nosology in DSM-5.1. *Psychopathology* 53 179–188. 10.1159/000507589 32369820

[B6] Bach B. Sellbom M. Kongerslev M. Simonsen E. Krueger R. F. Mulder R. (2017). Deriving ICD-11 personality disorder domains from DSM-5 traits: initial attempt to harmonize two diagnostic systems. *Acta Psychiatr. Scand*. 136 108–117. 10.1111/acps.12748 28504853

[B7] Bagby R. M. Sellbom M. (2018). The validity and clinical utility of the personality inventory for DSM-5 response inconsistency scale. *J. Pers. Assess*. 100 398–405. 10.1080/00223891.2017.1420659 29432027

[B8] Bauchowitz A. U. Gonder-Frederick L. A. Olbrisch M. E. Azarbad L. Ryee M. Y. Woodson M. (2005). Psychosocial evaluation of bariatric surgery candidates: a survey of present practices. *Psychosom. Med*. 67 825–832. 10.1097/01.psy.0000174173.32271.0 16204445

[B9] Baumeister H. Härter M. (2007). Mental disorders in patients with obesity in comparison with healthy probands. *Int. J. Obes.* 31 1155–1164. 10.1038/sj.ijo.0803556 17264844

[B10] Chalopin S. Betry C. Coumes S. Wion N. Reche F. Arvieux C. (2020). Benefits and risks of bariatric surgery in patients with bipolar disorders. *Surg. Obes. Relat. Dis*. 16 798–805. 10.1016/j.soard.2020.02.010 32209316

[B11] Clarkin J. F. Caligor E. Sowislo J. F. (2020). An object relations model perspective on the alternative model for personality disorders (DSM-5). *Psychopathology* 53 141–148. 10.1159/000508353 32698184 PMC7949219

[B12] De Panfilis C. Cero S. Torre M. Salvatore P. Dall’Aglio E. Adorni A. (2006). Utility of the temperament and character inventory (TCI) in outcome prediction of laparoscopic adjustable gastric banding: preliminary report. *Obes. Surg*. 16 842–847. 10.1381/096089206777822278 16839480

[B13] Fabricatore A. N. Crerand C. E. Wadden T. A. Sarwer D. B. Krasucki J. L. (2006). How do mental health professionals evaluate candidates for bariatric surgery? Survey results. *Obes. Surg*. 16 567–573. 10.1381/096089206776944986 16687023

[B14] Frankenburg F. R. Zanarini M. C. (2006). Obesity and obesity-related illnesses in borderline patients. *J. Pers. Disord*. 20 71–80. 10.1521/pedi.2006.20.1.71 16563080

[B15] Gerlach G. Herpertz S. Loeber S. (2015). Personality traits and obesity: a systematic review. *Obes. Rev*. 16 32–63. 10.1111/obr.12235 25470329

[B16] Guisado J. A. Vaz F. J. Alarcón J. López-Ibor J. J. Jr. Rubio M. A. Gaite L. (2002). Psychopathological status and interpersonal functioning following weight loss in morbidly obese patients undergoing bariatric surgery. *Obes. Surg*. 12 835–840. 10.1381/096089202320995664 12568191

[B17] Heriseanu A. I. Hay P. Corbit L. Touyz S. (2020). Relating goal-directed behaviour to grazing in persons with obesity with and without eating disorder features. *J. Eat. Disord.* 8:48. 10.1186/s40337-020-00324-1 33014370 PMC7528325

[B18] Hopwood C. J. Wright A. G. Krueger R. F. Schade N. Markon K. E. Morey L. C. (2013). DSM-5 pathological personality traits and the personality assessment inventory. *Assessment* 20 269–285. 10.1177/1073191113486286 23610235

[B19] Insel T. Cuthbert B. Garvey M. Heinssen R. Pine D. S. Quinn K. (2010). Research domain criteria (RDoC): toward a new classification framework for research on mental disorders. *Am. J. Psychiatry* 167 748–751. 10.1176/appi.ajp.2010.09091379 20595427

[B20] Kakoschke N. Aarts E. Verdejo-García A. (2019). The cognitive drivers of compulsive eating behavior. *Front. Behav. Neurosci*. 12:338. 10.3389/fnbeh.2018.00338 30705625 PMC6344462

[B21] Keeley J. W. Webb C. Peterson D. Roussin L. Flanagan E. H. (2016). Development of a response inconsistency scale for the personality inventory for DSM-5. *J. Pers. Assess*. 98 351–359. 10.1080/00223891.2016.1158719 27049169

[B22] Kerber A. Schultze M. Müller S. Rühling R. M. Wright A. G. Spitzer C. (2020). Development of a short and ICD-11 compatible measure for DSM- 5 maladaptive personality traits using ant colony optimization algorithms. *Assessment* 28:1073191120971848. 10.31234/osf.io/rsw54 PMC8866743 33371717

[B23] Kernberg O. F. (1984). *Severe Personality Disorders: Psychotherapeutic Strategies.* New Haven, CT: Yale University Press.

[B24] Kinzl J. F. Schrattenecker M. Traweger C. Mattesich M. Fiala M. Biebl W. (2006). Psychosocial predictors of weight loss after bariatric surgery. *Obes. Surg*. 16 1609–1614. 10.1381/096089206779319301 17217637

[B25] Kotov R. Krueger R. F. Watson D. Achenbach T. M. Althoff R. R. Bagby R. M. (2017). The Hierarchical Taxonomy of Psychopathology (HiTOP): a dimensional alternative to traditional nosologies. *J. Abnorm. Psychol*. 126 454–477. 10.1037/abn0000258 28333488

[B26] Krueger R. F. Derringer J. Markon K. E. Watson D. Skodol A. E. (2012). Initial construction of a maladaptive personality trait model and inventory for DSM-5. *Psychol. Med*. 42 1879–1890. 10.1017/S0033291711002674 22153017 PMC3413381

[B27] Larsen J. K. Geenen R. Maas C. de Wit P. van Antwerpen T. Brand N. (2004). Personality as a predictor of weight loss maintenance after surgery for morbid obesity. *Obes. Res*. 12 1828–1834. 10.1038/oby.2004.227 15601979

[B28] Leombruni P. Pierò A. Dosio D. Novelli A. Abbate-Daga G. Morino M. (2007). Psychological predictors of outcome in vertical banded gastroplasty: a 6 months prospective pilot study. *Obes. Surg*. 17 941–948. 10.1007/s11695-007-9173-4 17894155

[B29] Livhits M. Mercado C. Yermilov I. Parikh J. A. Dutson E. Mehran A. (2012). Preoperative predictors of weight loss following bariatric surgery: systematic review. *Obes. Surg*. 22 70–89. 10.1007/s11695-011-0472-4 21833817

[B30] Lowmaster S. E. Hartman M. J. Zimmermann J. Baldock Z. C. Kurtz J. E. (2020). Further validation of the response inconsistency scale for the Personality inventory for DSM-5. *J. Pers. Assess*. 102 743–750. 10.1080/00223891.2019.1674320 31625765

[B31] Marek R. J. Ben-Porath Y. S. Heinberg L. J. (2016). Understanding the role of psychopathology in bariatric surgery outcomes. *Obes. Rev*. 17 126–141. 10.1111/obr.12356 26783067

[B32] Marek R. J. Ben-Porath Y. S. Sellbom M. McNulty J. L. Heinberg L. J. (2015a). Validity of minnesota multiphasic personality inventory-2-restructured form (MMPI-2-RF) scores as a function of gender, ethnicity, and age of bariatric surgery candidates. *Surg. Obes. Relat. Dis*. 11 627–634. 10.1016/j.soard.2014.10.005 25487292

[B33] Marek R. J. Tarescavage A. M. Ben-Porath Y. S. Ashton K. Merrell Rish J. Heinberg L. J. (2015b). Using presurgical psychological testing to predict 1-year appointment adherence and weight loss in bariatric surgery patients: predictive validity and methodological considerations. *Surg. Obes. Relat. Dis*. 11 1171–1181. 10.1016/j.soard.2015.03.020 26003898

[B34] Marek R. J. Ben-Porath Y. S. van Dulmen M. H. M. Ashton K. Heinberg L. J. (2017). Using the presurgical psychological evaluation to predict 5-year weight loss outcomes in bariatric surgery patients. *Surg. Obes. Relat. Dis*. 13 514–521. 10.1016/j.soard.2016.11.008 28089590

[B35] Moore C. F. Sabino V. Koob G. F. Cottone P. (2017). Pathological overeating: emerging evidence for a compulsivity construct. *Neuropsychopharmacology* 42 1375–1389. 10.1038/npp.2016.269 27922596 PMC5436113

[B36] Morey L. C. (2017). Development and initial evaluation of a self-report form of the DSM-5 level of personality functioning scale. *Psychol. Assess*. 29 1302–1308. 10.1037/pas0000450 28240933

[B37] Olajide K. Munjiza J. Moran P. O’Connell L. Newton-Howes G. Bassett P. (2018). Development and psychometric properties of the Standardized Assessment of Severity of Personality Disorder (SASPD). *J. Pers. Disord*. 32 44–56. 10.1521/pedi_2017_31_285 28513349

[B38] Oltmanns J. R. Widiger T. A. (2018). A self-report measure for the ICD-11 dimensional trait model proposal: the personality inventory for ICD-11. *Psychol. Assess*. 30 154–169. 10.1037/pas0000459 28230410 PMC5930359

[B39] Oltmanns J. R. Rivera Rivera J. Cole J. Merchant A. Steiner J. P. (2020). Personality psychopathology: longitudinal prediction of change in body mass index and weight post-bariatric surgery. *Health Psychol*. 39 245–254. 10.1037/hea0000842 31944798 PMC7021354

[B40] Petry N. M. Barry D. Pietrzak R. H. Wagner J. A. (2008). Overweight and obesity are associated with psychiatric disorders: results from the national epidemiologic survey on alcohol and related conditions. *Psychosom. Med*. 70 288–297. 10.1097/PSY.0b013e3181651651 18378873

[B41] Pincus A. L. Cain N. M. Halberstadt A. L. (2020). Importance of self and other in defining personality pathology. *Psychopathology* 53 133–140. 10.1159/000506313 32114579

[B42] R Core Team (2021). *R: A Language and Environment for Statistical Computing.* Vienna: R Foundation for Statistical Computing.

[B43] Riegel K. D. Ksinan A. J. Samankova D. Preiss M. Harsa P. Krueger R. F. (2018). Unidimensionality of the personality inventory for DSM-5 facets: evidence from two Czech-speaking samples. *Personal. Ment. Health* 12 281–297. 10.1002/pmh.1423 29952078

[B44] Riegel K. D. Preiss M. Ksinan A. J. Michalec J. Samankova D. Harsa P. (2017). Psychometric properties of the Czech version of the personality inventory for DSM-5: internal consistency, validity and discrimination capacity of the measure. *Czechoslov. Psychol.* 61 128–143.

[B45] Rowe J. L. Downey J. E. Faust M. Horn M. J. (2000). Psychological and demographic predictors of successful weight loss following silastic ring vertical stapled gastroplasty. *Psychol. Rep*. 86 1028–1036. 10.2466/pr0.2000.86.3.1028 10876361

[B46] Sansone R. A. Schumacher D. Wiederman M. W. Routsong-Weichers L. (2008). The prevalence of binge eating disorder and borderline personality symptomatology among gastric surgery patients. *Eat. Behav*. 9 197–202. 10.1016/j.eatbeh.2007.08.002 18329598

[B47] Shen S. C. Lin H. Y. Huang C. K. Yen Y. C. (2016). Adherence to psychiatric follow-up predicts 1-year BMI loss in gastric bypass surgery patients. *Obes. Surg*. 26 810–815. 10.1007/s11695-015-1821-5 26208411

[B48] Somma A. Borroni S. Kelley S. E. Edens J. F. Fossati A. (2018). Further evidence for the validity of a response inconsistency scale for the Personality inventory for DSM-5 in Italian community-dwelling adolescents, community-dwelling adults, clinical adults. *Psychol. Assess*. 30 929–940. 10.1037/pas0000547 29565615

[B49] Tsushima W. T. Bridenstine M. P. Balfour J. F. (2004). MMPI-2 scores in the outcome prediction of gastric bypass surgery. *Obes. Surg*. 14 528–532. 10.1381/096089204323013550 15130232

[B50] Voon V. Morris L. S. Irvine M. A. Ruck C. Worbe Y. Derbyshire K. (2015). Risk-taking in disorders of natural and drug rewards: neural correlates and effects of probability, valence, and magnitude. *Neuropsychopharmacology* 40 804–812. 10.1038/npp.2014.242 25270821 PMC4305336

[B51] Walfish S. Vance D. Fabricatore A. N. (2007). Psychological evaluation of bariatric surgery applicants: procedures and reasons for delay or denial of surgery. *Obes. Surg*. 17 1578–1583. 10.1007/s11695-007-9274-0 18000719

[B52] World Health Organization [WHO] (2019). *ICD-11 Clinical Descriptions and Diagnostic Guidelines for Mental and Behavioural Disorders.* Geneva: World Health Organization.

[B53] Zimmermann J. Kerber A. Rek K. Hopwood C. J. Krueger R. F. (2019). A brief but comprehensive review of research on the Alternative DSM-5 model for personality disorders. *Curr. Psychiatry Rep*. 21:92. 10.1007/s11920-019-1079-z 31410586

[B54] Zimmermann J. Müller S. Bach B. Hutsebaut J. Hummelen B. Fischer F. (2020). A common metric for self-reported severity of personality disorder. *Psychopathology* 53 168–178. 10.1159/000507377

